# Integrative Analysis Reveals Across-Cancer Expression Patterns and Clinical Relevance of Ribonucleotide Reductase in Human Cancers

**DOI:** 10.3389/fonc.2019.00956

**Published:** 2019-10-04

**Authors:** Yongfeng Ding, Tingting Zhong, Min Wang, Xueping Xiang, Guoping Ren, Zhongjuan Jia, Qinghui Lin, Qian Liu, Jingwen Dong, Linrong Li, Xiawei Li, Haiping Jiang, Lijun Zhu, Haoran Li, Dejun Shen, Lisong Teng, Chen Li, Jimin Shao

**Affiliations:** ^1^Department of Pathology & Pathophysiology, and Cancer Institute of the Second Affiliated Hospital, Zhejiang University School of Medicine, Hangzhou, China; ^2^Key Laboratory of Disease Proteomics of Zhejiang Province, Key Laboratory of Cancer Prevention and Intervention of China National Ministry of Education, Research Center for Air Pollution and Health, Zhejiang University School of Medicine, Hangzhou, China; ^3^Key Laboratory of Precision Diagnosis and Treatment for Hepatobiliary and Pancreatic Tumor of Zhejiang Province, Department of Medical Oncology, Department of Pathology, The First Affiliated Hospital, Zhejiang University School of Medicine, Hangzhou, China; ^4^Department of Pathology, Sir Run Run Shaw Hospital, Zhejiang University School of Medicine, Hangzhou, China; ^5^Department of Human Genetics, Zhejiang University School of Medicine, Hangzhou, China; ^6^Discovery Biochemistry, Kymera Therapeutics, Cambridge, MA, United States; ^7^Southern California Permanente Medical Group, Department of Pathology, Downey Medical Center, Downey, CA, United States

**Keywords:** cancer-omics, integrative analysis, ribonucleotide reductase, expression characteristics, clinical relevance, gene networks

## Abstract

Mining cancer-omics databases deepens our understanding of cancer biology and can lead to potential breakthroughs in cancer treatment. Here, we propose an integrative analytical approach to reveal across-cancer expression patterns and identify potential clinical impacts for genes of interest from five representative public databases. Using ribonucleotide reductase (RR), a key enzyme in DNA synthesis and cancer-therapeutic targeting, as an example, we characterized the mRNA expression profiles and inter-component associations of three *RR* subunit genes and assess their differing pathological and prognostic significance across over 30-types of cancers and their related subtypes. Findings were validated by immunohistochemistry with clinical tissue samples (*n* = 211) collected from multiple cancer centers in China and with clinical follow-up. Underlying mechanisms were further explored and discussed using co-expression gene network analyses. This framework represents a simple, efficient, accurate, and comprehensive approach for cancer-omics resource analysis and underlines the necessity to separate the tumors by their histological or pathological subtypes during the clinical evaluation of molecular biomarkers.

## Background

To date, an immense and increasing amount of cancer-omics data and associated clinical annotation has been produced and has become publicly available from diverse repositories. Such cancer-omics data resources include the Catalog of Somatic Mutations in Cancer (COSMIC) (1), the Cancer Genome Atlas (TCGA) (2), the International Cancer Genome Consortium (ICGC) (3), and the Cancer Proteome Atlas (TCPA) (4). In such efforts, researchers have collected and annotated enormous amounts of heterogeneous cancer genomic data and have also created some powerful data-mining tools such as Oncomine (5), cBio Cancer Genomics Portal (cBioPortal) (6), Kaplan-Meier plotter (KM-plotter) (7), and the Human Protein Atlas (HPA) (8). Although these resources have provided an unprecedent opportunity to deepen our understanding of cancer development and progression, the challenge continues to press on toward increasingly efficient and more effective data mining strategies that aim at the identification of key molecular targets for improving cancer treatment.

Ribonucleotide reductase (RR) has been identified as an important anticancer target and its inhibitors, alone or combined with other anticancer drugs, have been successfully used to control multiple solid and hematological malignancies (9–12). However, three genes coding for RR proteins are located in three different chromosomes and their expressions are both varied and diverse in different types of cancers and their histologic variants (9, 10). The whole-genome expressional landscape of different RR subunit genes during cancer development, as well as their underlying molecular regulatory mechanisms and potential clinical applications, still requires considerable clarification. This makes RR an ideal candidate example to use for demonstrating a more integrative analytical approach for the mining of cancer-omics databases.

In this study, we began by analyzing multiple publicly available cancer-omics databases for the expression profiles of a group of RR-related genes and their correlations with patient survival. Findings were further validated using clinical samples from cohorts of lung cancer patients. With this approach, we revealed the expressional patterns of three RR subunit genes and their associations with each other in common types of cancers in Oncomine and TCGA datasets. The differential expression and prognostic significance of the two types of RR (*RRM1-RRM2* and *RRM1-RRM2B*) were further validated in 211 cases of lung squamous cell carcinoma (LUSC) and lung adenocarcinoma (LUAD) patients. Finally, we performed a gene co-expression network analysis to further define possible roles and explore the underlying mechanisms of the *RRM2* and *RRM2B* genes in LUAD and LUSC tumors. Our study provides novel insight in understanding molecular mechanisms of RR in cancer development, which may promote precision RR-targeting for cancer treatment and provides a valuable data-mining approach that could be applied to any gene of interest.

## Materials and Methods

### Databases and Datasets for Integrative mRNA Expression Analysis

Normalized microarray data used for analysis of *RRM1, RRM2*, and *RRM2B* mRNA expressions across 20 types of common human cancers were downloaded from the Oncomine database (5). Expectation-Maximization (RSEM) normalized RNA-sequencing (RNA-seq) data and clinicopathologic data of 31 types of common cancers, including BLCA (*n* = 408), BRCA (*n* = 1,093), CESC (*n* = 304), COAD (*n* = 285), COADREAD (*n* = 379), ESCA (*n* = 184), GBM (*n* = 153), GBMLGG (*n* = 669), HNSC (*n* = 520), KICH (*n* = 66), KIPAN (*n* = 889), KIRC (*n* = 533), KIRP (*n* = 290), LAML (*n* = 173), LGG (*n* = 516), LIHC (*n* = 371), LUAD (*n* = 515), LUSC (*n* = 501), OV (*n* = 303), PAAD (*n* = 178), PCPG (*n* = 179), PRAD (*n* = 497), READ (*n* = 94), SARC (*n* = 259), SKCM (*n* = 470), STAD (*n* = 415), STES (*n* = 599), TGCT (*n* = 150), THCA (*n* = 501), THYM (*n* = 120), and UCEC (*n* = 176) were downloaded from TCGA via Firehose. TCGA, Oncomine, and KM Plotter datasets were used for pathological survival analyses of LUSC and LUAD.

### Patient Samples in ZJUC Cohort

A total of 211 surgically-excised tumor tissue samples from LUSC and LUAD patients (*n* = 97 and 114, respectively) were collected between July 2011 and October 2013 in three hospitals in Zhejiang, China, including the First and Second Affiliated Hospitals of Zhejiang University, and Zhejiang Cancer Hospital, and the cohort was named as ZJUC cohort in this study. Prior to the study, all patients gave their written informed consent to allow the tissue samples and clinical information to be used for scientific research. The inclusion criteria were defined as follows: (i) histologically diagnosed as primary LUSC or LUAD; (ii) underwent surgical resection as a primary treatment; (iii) full information available including clinicopathologic characteristics and follow-up information. Patients were excluded if they had incomplete or missing data regarding the American Joint Committee on Cancer (AJCC) staging, survival state, cause of death and survival time. The disease stages were classified based on the 7th edition of AJCC staging manual. This study was approved by the Ethics Committee of each participating hospitals.

### Immunohistochemistry (IHC) Assays

The 211 tissue samples were formalin-fixed and paraffin-embedded. The immunohistochemistry was conducted using an Envision Detection System (DAKO, Denmark) according to the manufacturer's instructions as described previously (13). We used the following commercial antibodies against *RRM1* (10526-1-AP, Proteintech, 1:500), *RRM2* (ab57653, Abcam, 1:200), and *RRM2B* (ab8105, Abcam, 1:500) for immunohistochemistry. PBS was used as a negative control.

To determine the score of each slide, at least eight individual fields at 200× were selected, and 100 cancer cells were counted in each field. Cells with cytoplasmic and/or nuclear immunoreactivity of *RRM1, RRM2*, and *RRM2B* were considered positive. The immunostaining intensity was divided into five grades: 0, negative; 1, weak; 2, moderate; 3, strong; and 4, very strong. The proportion of positive-staining cells was also divided into five grades: 0, <5; 1, 6–25; 2, 26–50; 3, 51–75; and 4, >75%. The IHC scores were generated by multiplying the intensity score and the proportion score. To avoid observer bias, and for consistency, the value of immunostaining intensity and the percentage of positive-staining cells for all the slides were evaluated independently by two different well-trained observers blinded to the clinical data.

### Construction of Co-expressed Gene Networks for RR Subunit Genes in Lung Cancers

Based on RNA-seq data derived from TCGA, we estimated the correlations of gene sets tightly associated with RR subunit genes in different lung cancer types using Pearson's correlation coefficient. Gene-annotation enrichment analysis was next conducted using the DAVID bioinformatics resources version 6.8. Cytoscape 3.0 was used to visualize the topological molecular network structures that were composed of genes highly correlated to *RRM2B* with their Gene Ontology (GO) terms having *p* < 0.05 (14).

### Statistical Analysis

The definitions of overall survival (OS) and disease-free survival (DFS) followed recommended criteria (15). OS was defined as the interval between first pathological confirmation and death or the last date of follow-up. DFS was defined as the time from random assignment to recurrence, second primary cancer, or death with evidence of disease progression. Within a specific cohort, patients were divided into two groups by the median value of each gene in all samples. Associations with OS were examined using univariate Cox proportional hazard regression models. Survival curves were constructed using the Kaplan–Meier method and compared using the log-rank test. The correlation coefficients across individual gene expressions were calculated using Pearson's correlation coefficient in R 3.1.2 software package. Student *t* tests were used to compare continuous data. The One-way ANOVA was used to compare means of two or more samples, with the Least Significant Difference (LSD) test used for pair-wise comparisons of different groups. The Kaplan-Meier plot was drawn using GraphPad Software (version 6.0, USA). All tests were two tailed, retaining *P* < 0.05. Statistical analysis was performed using SAS v9.4 (SAS Institute, Inc., Cary, NC, USA).

## Results

### Development of an Integrative Analytic Approach for the Expression Pattern and Clinical Relevance

**Figure 1:**
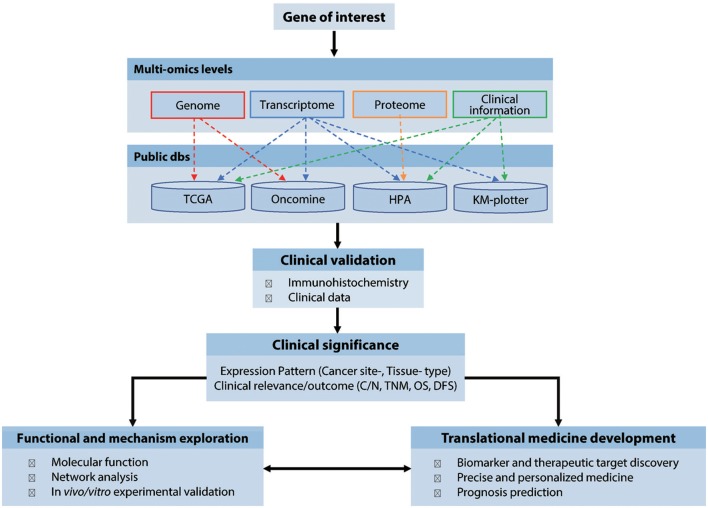
Workflow of the cancer-omics data based integratively analytic strategy. dbs, databases; N/C, Normal vs. Cancerous tissues; TNM, tumor, node, and metastasis classification; OS, Overall survival; DFS, Disease-free survival.

We propose a stepwise analytical workflow to investigate the expression patterns and clinical relevance of virtually any genes of interest related to the malignancies of human cancers (Figure 1). This firstly involves collection and integration of genomics, transcriptomics, proteomics, and clinical information from multiple publicly available cancer-omics databases, including COSMIC, TCGA, Oncomine, HPA, and Kaplan-Meier plotter. This data is then validated by routine immunochemical examination of clinical cancer samples collected from multiple archived tissue banks. Finally, the validated results are further explored for their biological relevance, underlying pathological mechanisms, and potential application in diagnosis, treatment, and prevention of cancers. In this manuscript, a multi-subunit enzyme, human RR, was used as an example to demonstrate the use, application and efficiency of this method.

### Expression Profiles of *RRM1, RRM2*, and *RRM2B* in Common Types of Human Cancers

**Figure 2:**
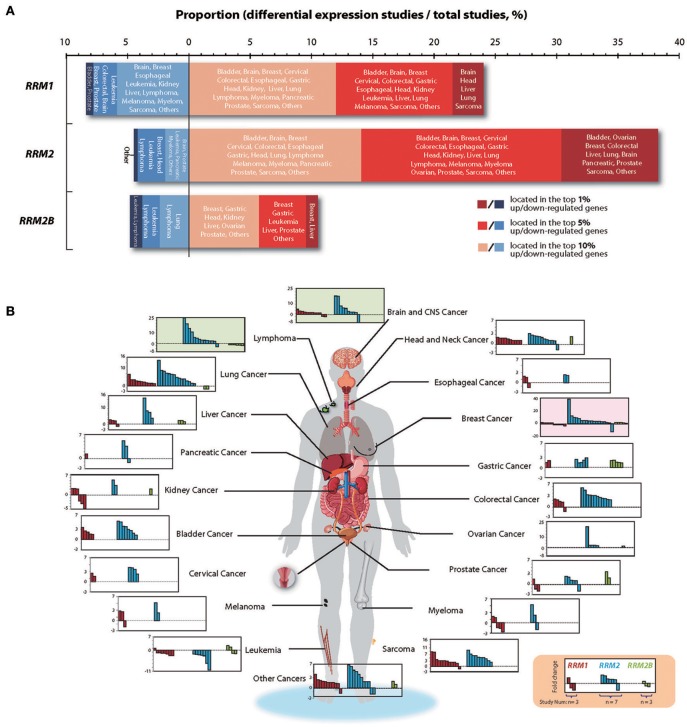
*RRM1, RRM2*, and *RRM2B* gene expression profiles in 20 common types of cancers. **(A)** mRNA expression data of *RRM1, RRM2*, and *RRM2B* genes in 20 common human cancers across all studies retrieved from Oncomine (version 4.5, search completed on Mar, 2019). DEGs were determined with the screening criteria “concept filter = cancers vs. normal, data type = mRNA, *P* < 0.05.” The x-axis indicated the proportion of studies with differentially expressed *RRM1, RRM2*, and *RRM2B* in all studies according the criteria. Dark red, red, and brick red colors marked the cancers in which the gene of interest had a gene rank of the top 1, 5, or 10% in the elevated-expression of the DEGs, respectively (Gene rank: genes are ranked by the *p*-value, for example, a gene rank ≤ 10% represents that its *p*-value lists in top 10% of the up-regulated or down-regulated ones). In contrast, dark blue, blue, and light blue colors marked the cancers in which the interested gene had a *p*-value rank of the top 1, 5, and 10% in the decreased-expression of the DEGs, respectively. **(B)** The visualization of fold change (FC) for *RRM1, RRM2*, and *RRM2B* mRNA expressions in the studies screened out (Details shown in Supplemental File 1). The FC values of overexpressed genes were calculated from tumor-/normal-tissues expression levels, while those for under-expressed genes were calculated from normal-/tumor-tissue expression levels. The human body parts diagram was adapted and modified from Robinson et al. (16).

To obtain mRNA expression profiles of three RR subunit genes in the 20 malignancy types, including both solid and blood malignancies, we systematically analyzed all the relevant studies (studies 358, 355 and 209 for *RRM1, RRM2*, and *RRM2B*, respectively) collected from Oncomine version 4.5, with the search completed on 15 Mar 2019 (Figure 2). We used following parameters as the filtering criteria to identify differentially expressed genes (DEGs) between cancer and normal tissues: concept filter = cancers vs. normal; data type = mRNA, *p* < 0.05, and gene rank being ≤10% (in which the genes are ranked by their *p*-value and a gene rank ≤10% represents those genes with their *p*-value listed in the top 10% of the up- or down-regulated genes). We found that the mRNA expression levels of *RRM1, RRM2*, and *RRM2B* ranked in the top 10% of the up-regulated DEGs in 24.0% (86 of 358), 38.3% (136 of 355), and 10.5% (22 of 209) of the identified cancer studies, respectively. In contrast, *RRM1, RRM2*, and *RRM2B* were ranked in the top 10% of the down-regulated DEGs in only 8.4% (30 of 358), 4.5% (16 of 355), and 4.8% (10 of 209) of the cancer studies, respectively. Figure 2A represents the global distribution of *RRM1, RRM2*, and *RRM2B* gene expressions across the different types of Oncomine cancer studies. Figure 2B presents the fold changes (FC, cancer vs. normal tissue) of mRNA expression of *RRM1, RRM2*, and *RRM2B* genes in each different type of the analyzed cancers. Obviously, *RRM2* gene expression levels were higher than that of other two subunits in almost all the studied cancer types, especially in brain and Central Nervous System (CNS) cancers, lymphomas, lung cancer, bladder cancer, head and neck cancer, breast cancer, colorectal cancer, and sarcomas.

### Associations of *RRM1, RRM2*, and *RRM2B* Expression in Common Types of Human Cancers

**Figure 3:**
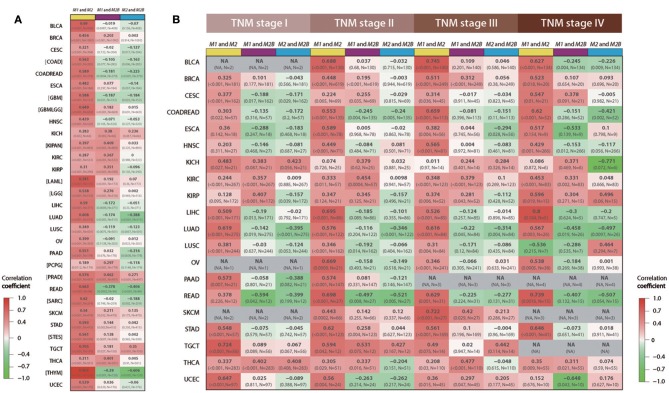
Associations among *RRM1, RRM2*, and *RRM2B* mRNA expressions in 31 types of human cancer. All data were retrieved from TCGA RNA-seq datasets via Firehose and analyzed using Spearman's correlation coefficient. Each cell of the figure contains the correlation coefficient (upper line), *p*-value and sample size (lower line). The cells were color-coded, red for positive correlations and green for negative correlations. Three different color bars at the top of each column of cells represented the association between *RRM1* and *RRM2* (yellow), *RRM1*, and *RRM2B* (purple), and *RRM2* and *RRM2B* (blue), respectively. **(A)** The overall mRNA expression relevance of the three RR subunits in 31 cancer types. **(B)** mRNA expression relevance of the three RR subunits in different TNM stages. NA, represents that the number of cases is <5; Brackets represent that the corresponding information about the TNM stages is not available.

To further study associations of *RRM1, RRM2*, and *RRM2B* expression in different cancer types and TNM stages, we downloaded and analyzed the RNA-seq and clinicopathologic data of 31 types of cancer from TCGA. Using the Spearman's correlation coefficient analysis, the consistent positive correlations were observed between *RRM1* and *RRM2* expression in all 31 types of cancer (Figure 3A). This consistency remained after subdividing patients into different TNM stages across almost all types of cancer (Figure 3B). In contrast, the associations between the expression of *RRM1* and *RRM2B* genes were relatively weak and variable.

### Expression Patterns of *RRM1, RRM2*, and *RRM2B* in Different Pathological Subtypes and TNM Stages of Lung Cancer

**Figure 4:**
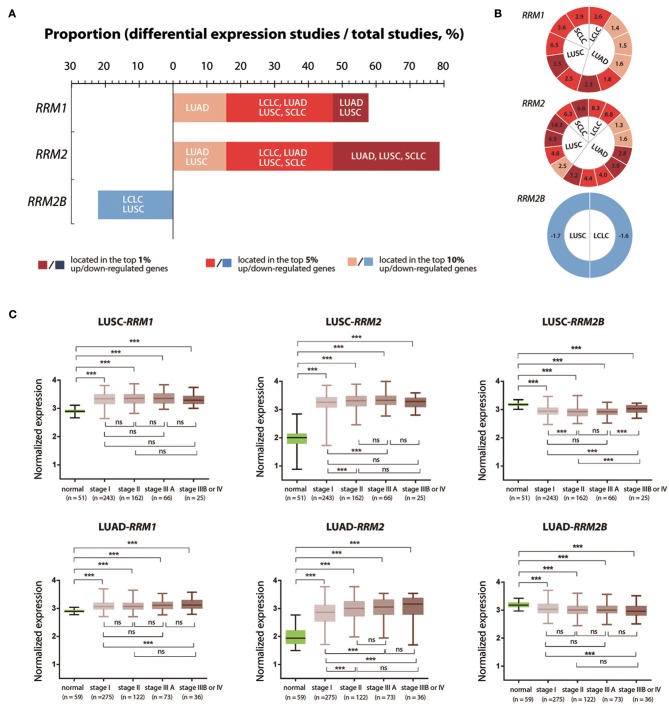
Expression changes of *RRM1, RRM2*, and *RRM2B* in different pathological types and different TNM stages of lung cancer. **(A)** mRNA expression data of *RRM1, RRM2*, and *RRM2B* in different pathological types of lung cancers across all studies were retrieved from Oncomine. DEGs were determined according to the above screening criteria. The x-axis indicated the proportion of studies with differentially expressed *RRM1, RRM2*, and *RRM2B* in all lung cancer studies. Dark red, red, and brick red colors mark the cancers in which the gene of interest had a gene rank of top 1, 5, or 10% among the elevated expression genes, respectively (Gene rank: genes are ranked by the *p*-value, for example, a gene rank ≤ 10% represents that its *P*-value lists in top 10% of the up-regulated or down-regulated genes). In contrast, dark blue, blue, and light blue colors mark the cancers in which the gene of interest had a gene rank of top 1, 5, and 10% among the decreased-expressed genes, respectively. **(B)** The visualization of fold change (FC) values for *RRM1, RRM2*, and *RRM2B* mRNA expressions in the studies of different pathological types of lung cancer. The number in each cell represents a FC value from one study. The definition of cell colors is the same as that in **(A)**. The FC value of an overexpressed gene was calculated from tumor-/normal-tissue expression levels; and the under-expressed gene was calculated from normal-/tumor-tissue expression levels. **(C)** The mRNA expression of *RRM1, RRM2*, and *RRM2B* in the different TNM stages of LUSC and LUAD. The RNA-seq and clinicopathological information of LUSC and LUAD patients were downloaded from TCGA through cBioPortal. Five groups of data (Para-cancerous tissues, TNM I, II, IIIA, and IIIB/IV cancer tissues) were analyzed using one-way ANOVA with LSD test for pair-wise comparisons of different groups. ***P < 0.05; ns, not significant.

To further characterize the expression pattern of *RRM1, RRM2*, and *RRM2B* in different pathological types and stages of cancer, we chosen lung cancer as a representative cancer type as it is the leading cause of cancer death all over the world. We performed differentially expressed genes (DEGs) analyses with the above filtering criteria using the Oncomine data. We found that the mRNA expression of *RRM1, RRM2*, and *RRM2B* genes were up-regulated in 57.9% (11 of 19), 78.9% (15 of 19), and 0% (0 of 9), respectively, of the Oncomine cancer studies covering the four subtypes of LUSC, LUAD, large cell lung carcinoma (LCLC), and small cell lung carcinoma (SCLC) (Figure 4A). In contrast, no study showed down-regulated expression of *RRM1* (0 of 19) or *RRM2* (0 of 19) in all subtypes of lung cancer, 22.2% (2 in 9) of the Oncomine studies demonstrated a down-regulated *RRM2B* expression in cases of LUSC and LCLC. Figure 4B presented the mRNA expression FC values of *RRM1, RRM2*, and *RRM2B* genes in different subtypes of lung cancer, which appeared quite variable across different types of lung cancer and even varied in the same type of tumor from different studies.

We then analyzed the mRNA expression of three RR subunit genes in different progression stages of LUSC and LUAD, the two main pathological subtypes of NSCLC, using the RNA-seq data retrieved from TCGA. The expression of *RRM1* and *RRM2* genes in tumor tissues were both significantly increased in all TNM stages of LUSC and LUAD, compared with their corresponding adjacent normal lung tissue samples. In contrast, the *RRM2B* gene showed a significantly lower expression in the cancer tissues in all the TNM stages (Figure 4C). *RRM2* expression was significantly higher in stages II and IIIA of LUSC and LUAD as well as in stages IIIB/IV of LUAD compared with its expression in stage I tumors (*p* < 0.05). In addition, *RRM1* expression was also significantly higher in stages IIIB/IV LUAD than in stage I tumors (*p* < 0.05). Interestingly, *RRM2B* expression was significantly higher in stages IIIB/IV LUSC than in the stages I, II and IIIA tumors (*p* < 0.05), but was significantly lower in stages IIIB/IV LUAD than in stage I tumors (*p* < 0.05).

### Different Prognostic Impacts of *RRM1, RRM2*, and *RRM2B* Expression in the LUSC and LUAD Patients

**Figure 5:**
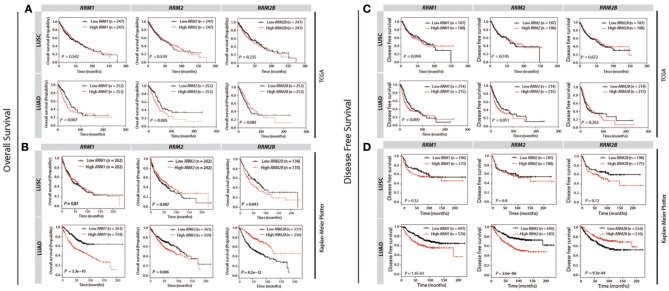
Survival curves for the patients of LUSC and LUAD against the expression levels of *RRM1, RRM2*, and *RRM2B* in TCGA and KM Plotter cohorts by using KM survival plotter analysis. The overall survival (OS) and disease free survival (DFS) against *RRM1, RRM2*, and *RRM2B* mRNA expressions in TCGA **(A,C)** and Kaplan-Meier Plotter **(B,D)** databases are shown, respectively. Vertical hash marks indicate points of censored data. For comparisons, the patients are dichotomized into two groups according to their median expression values.

To determine the prognostic values of three RR subunit expression in LUAD and LUSC patients, we analyzed the cancer survival data from TCGA and KM-plotter databases. In the TCGA database, higher *RRM1* and *RRM2* expression predicted a significantly shorter OS (*p* = 0.007 and 0.005 for *RRM1* and *RRM2*, respectively, Figure 5A) and DFS (*p* = 0.009 and 0.011 for *RRM1* and *RRM2*, respectively, Figure 5C) in the LUAD patients, compared with those with lower *RRM1* and *RRM2* expression. This correlation was not observed in the LUSC patients. Similarly, in the KM-plotter database, higher *RRM1* and *RRM2* expressions were also significantly associated with a shorter OS (*P* = 3.3e−10 and 0.006 for *RRM1* and *RRM2*, respectively, Figure 5B) and DFS (*P* = 1.1e−03 and 2.6e−06 for *RRM1* and *RRM2*, respectively, Figure 5D) in the LUAD patients, but not in the LUSC patients. The poor prognosis of high *RRM1* and *RRM2* expressions in LUAD patients was also observed in two additional independent studies (9, 10) from Oncomine (Figure S1).

Interestingly, we observed that a higher *RRM2B* expression was associated with a significantly better OS (*P* = 8.2e−12) and DFS (*P* = 9.3e−04) of the LUAD patients in the KM-plotter cohort (Figures 5B,D). In contrast, a higher *RRM2B* expression predicted a shorter OS (*P* = 0.043) and a poor-trend of DFS (*P* = 0.12) in LUSC patients. The differential prognostic implication of *RRM2B* expression in the LUSC and LUAD patients appeared to be weaker in the TCGA dataset when the median expression was selected as a cutoff point (Figure 5). However, if the optimal expression was used as a cutoff point, the cancer survivals predicted by *RRM2B* also trended differentially in the LUAD and LUSC patients, similar to the findings in the KM-plotter cohort (Figure S5). These results showed that *RRM2B* had opposite prognostic effects in the patients with LUAD and LUSC, which suggested that the *RRM2B* may function differently in LUAD and LUSC.

### Validation of the Protein Expression Patterns and Clinical Relevance of *RRM1, RRM2*, and *RRM2B* in the LUSC and LUAD Patients Using Immunohistochemistry and Survival Studies

To validate the potential implication of *RRM1, RRM2*, and *RRM2B* expression in pathology and prognosis of NSCLC, 211 surgically excised tumor specimens were independently collected from the patients with LUSC and LUAD (*n* = 97 and 114, respectively) from three academic hospitals affiliated to Zhejiang University in China (named the ZJUC cohort hereafter). The demographics and clinicopathological characteristics of the ZJUC cohort are described in Table S1. Among them, 72 percent of the ZJUC cohort were male with a mean age of 60.8 ± 9.6 years, and a median follow-up period of 45 months.

**Figure 6:**
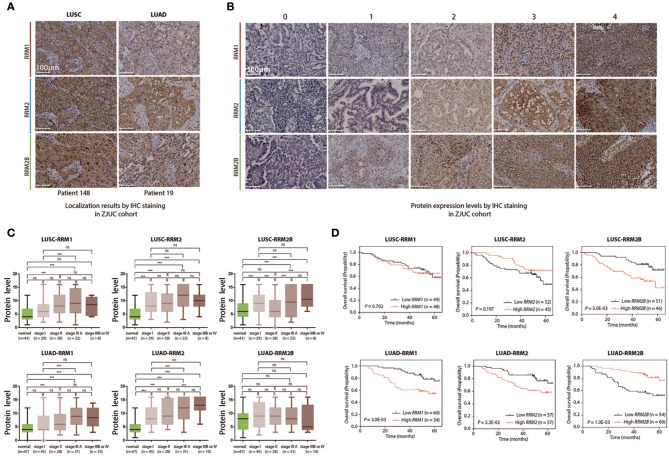
Clinical relevance analyses of *RRM1, RRM2*, and *RRM2B* protein expression levels in LUSC and LUAD patients including immunohistochemistry staining and follow-up studies. **(A)** Localization of *RRM1, RRM2*, and *RRM2B* by IHC staining in ZJUC cohort. **(B)** Protein expression levels by IHC staining in the ZJUC cohort. **(C)** Association between *RRM1, RRM2*, and *RRM2B* protein expression levels and prognosis of LUSC and LUAD patients. ***P < 0.001. **(D)** The protein expression of *RRM1, RRM2*, and *RRM2B* in the different TNM stages of LUSC and LUAD in the ZJUC cohort. IHC staining of *RRM1, RRM2*, and *RRM2B* proteins in the clinical tissue samples of LUSC and LUAD patients in the ZJUC cohort (97 LUSC and 114 LUAD). IHC staining was evaluated with a score range from 0 to 16. See Method for scoring criteria.

All specimens were examined by immunohistochemical (IHC) staining for three RR subunit expression. The results showed that *RRM1* and *RRM2* protein mainly localized in the cytosol while *RRM2B* protein was found in both the cytosol and nucleoplasm of LUSC and LUAD tumor cells (Figure 6A). This was consistent with the subcellular localization observed in an immunofluorescent study of three cancer cell lines (A-431, U-2 OS and U-251 MG) in HPA (Figure S2). Representative IHC pictures of three RR subunit protein expression in the NSCLC tissue samples were shown in Figure 6B. *RRM1* and *RRM2* protein expressions were significantly increased in both LUSC and LUAD cancer cells as compared to the adjacent normal lung tissues (Figure 6C). *RRM2* protein expression was significantly higher in the stage IIIA LUSC or LUAD tumors compared with those in the stage I tumors (*P* < 0.05). A similar pattern of *RRM2* protein expression was also observed in the stage IIIB/IV LUAD tumors compared to their stage I counterparts (*P* < 0.05). *RRM1* expression was also significantly higher in the stage IIIA LUSC and LUAD compared to the stage I tumors (*P* < 0.05). Interestingly, while *RRM2B* expression was also higher in the LUSC tumors, as compared to their adjacent normal lung tissues, and was higher in the stage IIIB/IV tumors than the stage II ones (*P* < 0.05), *RRM2B* expression appeared to be decreased in LUAD tumors although this level was not statistically significant.

Follow-up and survival analyses of ZJUC cohort patients suggested that higher *RRM1* and *RRM2* protein levels were associated with a significantly shorter OS in the LUAD patients (*P* = 0.003 and 0.023 for *RRM1* and *RRM2*, respectively), but not in the LUSC patients (*P* > 0.05). Higher *RRM2B* protein level was associated with a significantly shorter OS (*P* = 0.005) in the LUSC patients but a significantly prolonged OS (*P* = 0.001) in the LUAD patients (Figure 6D). These results were similar with what were obtained from data-mining (Figure 5). In conclusion, the IHC analysis of RR subunit protein expressions and the associated survival study in the ZJUC cohort of lung cancer patients demonstrated the usefulness and relevance of our bioinformatic analysis through data mining of multiple cancer-omics databases.

### Different Co-expression Gene Networks of *RRM2B* From *RRM2* in LUSC and LUAD

**Figure 7:**
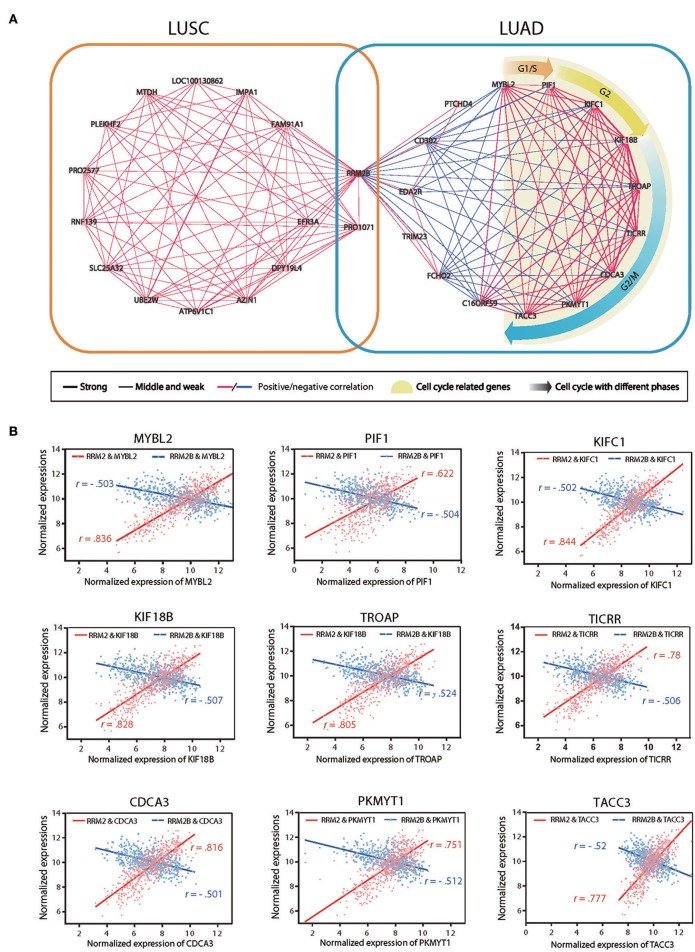
Gene co-expression networks of *RRM2B* in LUSC and LUAD. **(A)** Two co-expressed gene networks of highly correlated genes with *RRM2B* in LUSC (left panel) and LUAD (right) were constructed using Pearson's correlation coefficient (CC) analysis. Green nodes represent GO terms for gene function, pink nodes denote correlated genes, and lines connect paired co-expressed genes (blue for negative correlation and red for positive correlation). Three correlation intensities (strong: |CC| > 0.9; middle: 0.7 ≤ |CC| ≤ 0.9; weak: |CC| < 0.7) are represented by different line thickness. **(B)** The mRNA expression associations of *RRM2* or *RRM2B* with 10 *RRM2B* highly co-expressed genes (*MYBL2, PIF1, KIFC1, KIF18B, TROAP, TICRR, CDCA3, PKMYT1*, and *TACC3*) participating in the regulation of the cell cycle in LUAD. R: Pearson correlation coefficient.

To uncover the underlying mechanisms of the opposite prognostic effects of *RRM2B* expression in the cases of LUSC as opposed to LUAD, we performed a co-expression gene network analysis of *RRM2B* in these two subtypes of NSCLCs based on RNA-seq data from TCGA. We used the Pearson's correlation coefficient (CC) with around 0.5 as a cutoff, to identify the *RRM2B*-associated genes. The resulting networks consisted of 30 genes, 14 in LUSC and 16 in LUAD, with one gene in common. These genes were connected via 166 expressional interactions. The positive or negative correlations and the different correlation intensities are visualized using Cytoscape 3.0 software (Figure 7A and Table S2). Significantly, the results showed two distinct panels of co-expressed genes connected by *RRM2B* between LUAD and LUSC, with only one overlapping gene.

A further functional enrichment analysis of two co-expression gene networks using DAVID bioinformatics resources version 6.8 found that the gene set negatively associated with *RRM2B* expression in LUAD was enriched together with the genes promoting cell cycle progression and malignant aggressiveness, such as those of cell cycle regulation related biological processes and DNA repair. In contrast, the gene set co-expressed with *RRM2B* in the LUSC consisted of the genes associated with protein destabilization and the ERAD pathway and were not the obligate participants of cell cycle regulation. These results suggested that *RRM2B* may play double- or multifaceted roles in different types of lung carcinomas using distinct molecular machineries which resulted in its opposite prognostic impacts on LUAD and LUSC. In addition, nine genes involved in cell cycle progression (*MYBL2, PIF1, KIFC1, KIF18B, TROAP, TICRR, CDCA3, PKMYT1*, and *TACC3*) were found reversely correlated with *RRM2B* expression, but tightly positive-correlated with *RRM2* expression, in LUAD (Figure 7B). This observation further suggests that the different associations of *RRM2B* and *RRM2* proteins with the members of cell cycle pathway in LUAD may be the underlying mechanism of the opposite prognostic effects of different RR subunits observed in patients with LUAD and LUSC.

## Discussion

Among the current publicly available cancer-omics databases, those genomics and transcriptomics platforms with data-mining tools and data integration functions have been widely used in cancer research and precision medicine. Table S3 shows some popular cancer-omics databases and their main features (1–8, 11, 17–19). However, considering the different specimen sources and the nature of cancer heterogeneity, significant amounts of the data needs to be collected and crossly validated. HPA provides global analysis of how cancer genomes are expressed at the protein level, but its application is limited by a small sample size and lack of quantification. More recently, with the advance of high-throughput single-cell genomics, the Human Cell Atlas (HCA) Project has made progress in defining distinctive molecular profiles of all the human cell types and functions (12) and may soon help promote a profound understanding of the human tumor cellular ecosystem. Although clinical application of cancer-omics is encouraging, a significant challenge remains in terms of how to more efficiently use these rapidly expanding big data sets from multiple sources. In this work, using the study of three RR subunit genes (*RRM1, RRM2*, and *RRM2B*) as an example, we proposed a straightforward approach for integrative analysis of multiple cancer omics databases of virtually any genes of interest, which, combined with clinical patients validation, can improve the definition of cancer gene expressional patterns to further elucidate their underlying mechanisms as well as facilitate their clinical application (Figure 1).

RR plays a key role in DNA synthesis and thus is essential for cell proliferation and the development of malignancy (20). In normal human cells, three RR subunit proteins form two-types of holoenzymes (*RRM1- RRM2* and *RRM1- RRM2B*) and their genes are separately distributed in three different chromosomes. Although *RRM2* and *RRM2B* are highly homologous in their gene sequences, their expressional levels and subcellular localizations are differently regulated in cells. The expression and degradation of *RRM2* is regulated in a cell cycle-dependent manner (21), while *RRM2B* is induced in response to DNA damage in a p53-dependent manner (22). Our IHC examination of NSCLC tumor tissue and immunofluorescent images of three cell lines in the HPA have demonstrated that both *RRM1* and *RRM2* are mainly expressed in the cytoplasm, whereas *RRM2B* is expressed both in the cytoplasm and the nucleus (Figure 6A and Figure S2). This is consistent with the notion that ribonucleotide reduction with *RRM2*-containing RR takes place in the cytoplasm and the dNTPs are then transported into the nucleus for DNA synthesis. Conversely, *RRM2B*-containing RR is more likely to remain in the nucleus, ready for the repair of genetic abnormalities which constantly occur in human cancer cells (23, 24).

In human cancers, the mutation rates of *RRM1, RRM2*, and *RRM2B* are all below 0.5%, based on the data from COSMIC and cBioPortal (Table S4). The primary form of mutation is a missense substitution, which appears to be randomly distributed. Only several mutants (e.g., *RRM1*^R499C/H^, *RRM2B*^P308L/S^, *RRM2B*^F323L^) have been identified more than three times. There is almost no copy number variation (CNV) of *RRM1* and *RRM2* genes in common types of cancers, whereas *RRM2B* displays CNV gain in more than 3% of breast cancers, liver cancers, stomach cancers and urinary tract cancers. In this study, we found that gene expression dysregulation might be the dominant characteristic of the multi-subunit enzyme RR in human cancers. The expression of three *RR* genes, especially *RRM1* and *RRM2*, increased significantly across common types of cancers (Figure 2). Typically, *RRM2* up-expression occurred in almost all malignancies, and the FCs of *RRM2* expression between cancer and normal tissues were much larger than those in either *RRM1* or *RRM2B*. The gene expression of *RR* in human cancers has been analyzed previously using Oncomine (last updated in Nov, 2013) (25). Here, we further analyzed up to 358, 355, and 209 Oncomine studies for the differential expression of *RRM1, RRM2*, and *RRM2B*, respectively (detail comparisons in Table S5). With approximately twice as many studies collected in recent years, we demonstrated a more extensive expressional landscape and increased characteristics of *RR* genes in common types of cancers. Some additional information was also included, such as the down-regulated expression of *RRM2B* in lung cancer, leukemia and lymphoma, and the down-regulation of *RRM1* expression in bladder, breast and prostate cancers. Importantly, for the first time, we revealed the correlations among *RRM1, RRM2*, and *RRM2B* expression during cancer development as well as their individual roles and collaborative relationships during cancer progression together with their prognosis with clinical tumor sample validation.

The differential expressional changes and possible roles of three RR components have been noted previously in different clinical cancer samples, including lung (26), digestive tract (13, 27, 28) and other cancers (29). Among the three RR subunit genes, *RRM2* has been proposed to be an oncoprotein (25, 30). Upregulation of *RRM2* has been associated with cancer cell proliferation, invasion and metastasis in multiple types of cancers (28, 29, 31, 32). In contrast, although *RRM1* expression was also found to be up-regulated in many types of cancers, its role in cancer development remains controversial. Consistent with the role of RR in DNA replication and repair, a high *RRM1* expression is known to be associated with a poor response to the DNA-damaging platinum drugs and to the *RRM1*-targeting drug gemcitabine, and thus led to poor outcomes in these cancer patients (33–35). However, several studies have also shown that a highly expressed *RRM1* might be associated with a better outcome for some cancer patients (36, 37), suggestive of a suppressor role of tumor initiation, invasion and metastasis. A recent study demonstrated that *RRM1* can negatively regulate ZRANB3 function when in the nucleus, leading to DNA-synthesis reduction (38). The role of *RRM2B* in tumors is also not clear (25, 30). For example, it has been reported that *RRM2B* expression was reversely associated with tumor metastasis and was correlated with a better survival in colorectal cancer patients (39, 40). *RRM2B* expression was also shown to be reversely associated with intrahepatic metastasis in hepatocellular carcinomas (41). Conversely, *RRM2B* expression has been previously reported to be positively related to the development of esophageal squamous cell carcinoma (27). In this study, by combining RNA-seq and clinicopathologic data from Oncomine and TCGA (Figures 2, 3), we found that expression of *RRM1* and *RRM2* genes were both significantly increased and correlated with higher TNM stages in most common types of cancers and demonstrated a tumor-promoting role for the *RRM1-RRM2* holoenzyme. In contrast, the expression of *RRM2B* in these cancers varied, and the correlations between the expression of *RRM1* and *RRM2B* were weak and variably dependent on cancer type. Notably, the expression of *RRM2* and *RRM2B* were much weaker or even reversely correlated in some types of cancers. These data suggested that expression of three *RR* genes, and thus the related enzyme activities, may be dysregulated in the cancer cells. They probably operated under different mechanisms related to aspects of cancer progression or patient survival in different cancer types and stages. Moreover, the differential expression pattern of three *RR* genes in different types of human cancers suggested that, in addition to forming a RR holoenzyme to implement the classical enzymatic function for cancer cell proliferation, each of the three component proteins may be individually recruited by cancer cells to play a different non-RR enzymatic role during cancer initiation and development.

The clinical significance of different RR expression in cancers have attracted much attention, especially in NSCLC (33, 42). However, discrepancies among different reports remains to be elucidated. For *RRM1*, some studies showed that high *RRM1* expression was associated with better survival in early stage NSCLC (26) or had poor prognosis in advanced NSCLC (43) or lung adenocarcinoma (44), while another study showed that *RRM1* protein expression had no significant predictive value for early NSCLC patients (45). For *RRM2*, some studies suggested that high expression of *RRM2* prognosticated a shorter overall survival for NSCLC patients (46, 47), while the others did not find any predictive value (26, 48). For *RRM2B*, while it was shown that high expression of *RRM2B* protein was a favorable prognostic factor in early NSCLC patients (49), other authors reported that *RRM2B* protein expression did not play a prognostic role in NSCLC patients with resected TNM stages I–III tumors (45, 50). Such controversies can be attributed into the following reasons: different expression detection means, sample selection bias and cancer heterogeneity. In this study, we revealed another important reason that there was no distinction of different pathological subtypes. LUSC arises in proximal airways and is more strongly associated with smoking and chronic inflammation than is LUAD, which arises more frequently in the distal airways. By integrative cancer-omics data analysis and clinical sample validation with IHC, we showed that differential expression of three RR components individually and collaboratively impact malignant progression and patient survival in LUSC and LUAD. In addition, we found that LUSC had higher *RRM1* and *RRM2* expression than LUAD, while *RRM2B* expressed lower in tumor tissues of LUSC than in those of LUAD (Figure S3). The results implied the different roles of RR subunits in LUSC and LUAD. Recently, Uhlen et al. found high expression of the Endoplasmic Reticulum Oxidoreductase 1 Alpha (ERO1A) gene was correlated with a bad prognosis in NSCLC (including LUSC and LUAD) (51). However, after distinguishing pathological subtypes, we also found opposite prognostic effects of a high expression of *ERO1A* in LUSC and LUAD (Figure S4). This further supported the necessity to separate the tumors by their histological or pathological subtypes during research and clinical evaluation of molecular biomarkers in NSCLC.

It was unanticipated that a higher expression of *RRM2B* was significantly associated with a shorter survival in LUSC but a more prolonged survival in LUAD, while higher expressed *RRM1* and *RRM2* predicted a poorer clinical outcome in LUAD (Figures 5, 6). By co-expression gene network analysis, distinct gene sets were revealed to be associated with *RRM2* and *RRM2B* in LUSC and LUAD, respectively (Figures 7A,B and Table S1). *RRM2B* expression was reversely correlated with cell cycle-promoting molecules in LUAD but not in LUSC, suggesting that *RRM2B* may directly or indirectly suppress cell cycle progression, hence cancer cell proliferation, in LUAD. DNA microarray analysis has shown that the gene set regulating cell-cycle progression was significantly enriched in p53R2 (the alias of *RRM2B*)-silenced human KB oropharyngeal carcinoma cells. *RRM2B* may suppress cancer cell proliferation partially by upregulation of p21 and downregulation of cyclin D1 in addition to playing a critical role in DNA repair (52). By contrast, *RRM2* expression was significantly associated with cell cycle-progression (Figure 7B), consistent with its role as a RR subunit to promote cancer cell proliferation. Thus, the different co-expressed gene networks are compatible with the opposite roles and different underlying mechanisms of *RRM2* and *RRM2B* in different types of cancers, which further stressed the importance of the rationale of the use of RR inhibitors in precision cancer medicine.

## Conclusions

Taken together, the systematic translation of cancer-omics data into the fields of tumor biology and cancer therapy remains challenging. Herein, we demonstrated an integrative analytical approach based on mining multiple cancer-omics databases to reveal across-cancer-type expression patterns and their impacts on clinical outcomes of any genes of interest, followed by validation of protein-levels in clinical cancer samples from multiple cancer centers. Using this technique, we depicted a landscape of expression and association of three RR components in different types of common cancers. We further demonstrated a more complex pattern of three RR components in different subtypes and stages of lung cancer and associated these with clinical survival and discussed the possible underlying mechanisms. While the extensive high-expression of RR components in common cancers suggests them to be the targets of a broad-spectrum anti-cancer therapies, the heterogeneous expression pattern of each component of RR in different types of cancers and individual cancer patient supports their important requirement for more specifically targeted aspects and increased precision in cancer therapy.

## Novelty and Impact

Herein, we developed an integrative analytical approach to reveal across-cancer expression patterns and identify potential clinical impacts for genes of interest from public databases. Using ribonucleotide reductase (RR) as an example, we characterized the expression profiles and inter-component associations of three RR subunit genes across over 30-types of cancers. In addition, we assessed and validated pathological and prognostic significance of RR in lung cancer and related pathological subtypes. Underlying mechanisms were further explored and discussed.

## Data Availability Statement

The datasets generated for this study are available on request to the corresponding author.

## Ethics Statement

The study was conducted according to the principles of the Declaration of Helsinki, and it was approved by the First and Second Affiliated Hospitals of Zhejiang University, and Zhejiang Cancer Hospital, Zhejiang, China (No. 2018-753-2). Written informed consents were obtained from all subjects enrolled in this study.

## Author Contributions

JS, CL, and LT designed and supervised the research. YD, CL, and TZ analyzed the data. YD, CL, MW, TZ, LL, and XL prepared the figures and tables. TZ, XX, GR, and ZJ constructed the IHC. YD, CL, and TZ wrote the manuscript. JS, CL, LT, YD, DS, HL, LZ, HJ, QLin, QLiu, and TZ revised the manuscript. All authors read and approved the final manuscript.

### Conflict of Interest

The authors declare that the research was conducted in the absence of any commercial or financial relationships that could be construed as a potential conflict of interest.

## Abbreviations

BLCA: Bladder urothelial carcinoma
BRCA: Breast invasive carcinoma
CESC: Cervical and endocervical cancers
COAD: Colon adenocarcinoma
COADREAD: Colorectal adenocarcinoma
DEGs: Differentially expressed genes
ESCA: Esophageal carcinoma
GBM: Glioblastoma multiforme
GBMLGG: Glioma
HNSC: Head and Neck squamous cell carcinoma
KICH: Kidney Chromophobe
KIPAN: Pan-kidney cohort (KICH+KIRC+KIRP)
KIRC: Kidney renal clear cell carcinoma
KIRP: Kidney renal papillary cell carcinoma
LAML: Acute Myeloid Leukemia
LCLC: large cell lung carcinoma
LGG: Brain Lower Grade Glioma
LIHC: Liver hepatocellular carcinoma
LUAD: Lung adenocarcinoma
LUSC: Lung squamous cell carcinoma
OV: Ovarian serous cystadenocarcinoma
PAAD: Pancreatic adenocarcinoma
PCPG: Pheochromocytoma and Paraganglioma
PRAD: Prostate adenocarcinoma
READ: Rectum adenocarcinoma
SARC: Sarcoma
SCLC: small cell lung carcinoma
SKCM: Skin Cutaneous Melanoma
STAD: Stomach adenocarcinoma
STES: Stomach and Esophageal carcinoma
TGCT: Testicular Germ Cell Tumors
THCA: Thyroid carcinoma
THYM: Thymoma
UCEC: Uterine Corpus Endometrial Carcinoma.
