# Supplementary material for: Integrative Analysis Reveals Across-Cancer Expression Patterns and Clinical Relevance of Ribonucleotide Reductase in Human Cancers
Source: Front Oncol. 2019 Oct 4;9:956. doi: 10.3389/fonc.2019.00956 (PMC6788385; doi:10.3389/fonc.2019.00956)

## Supplementary Figures

### Figure S1. Association between *RRM1* and *RRM2* mRNA expressions and overall survival in LUSC and LUAD patients from Oncomine. The data with LUSC were based on the study of Raponi et al [27] and the data with LUAD were based on the study of DirecotorsChallenge et al [28].


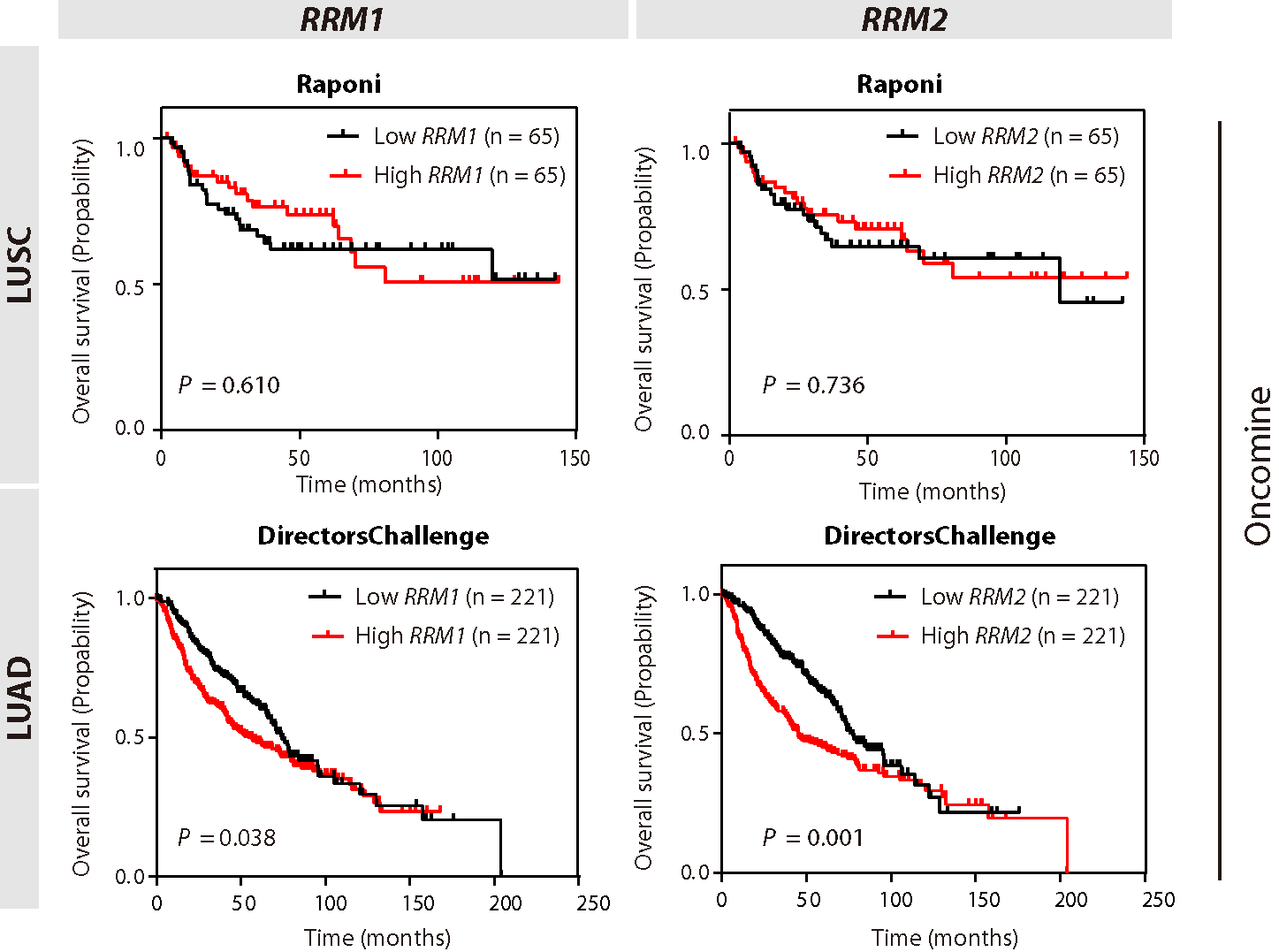


### Figure S2. Immunofluorescent subcellular localizations of RRM1, RRM2 and RRM2B proteins. Immunofluorescent subcellular localizations of RRM1, RRM2 and RRM2B in different human cancer cell lines were obtained from HPA website. A-431, U-2 OS, and U-251 MG represented epidermal carcinoma cell line, osteosarcoma cell line, and malignant glioma cell line, respectively. Green, immunofluorescent staining with the antibodies against RRM1 (CAB022093), RRM2 (HPA056994), or RRM2B (HPA028812); blue, nucleus staining with DAPI. Scalebar: 30 μm.


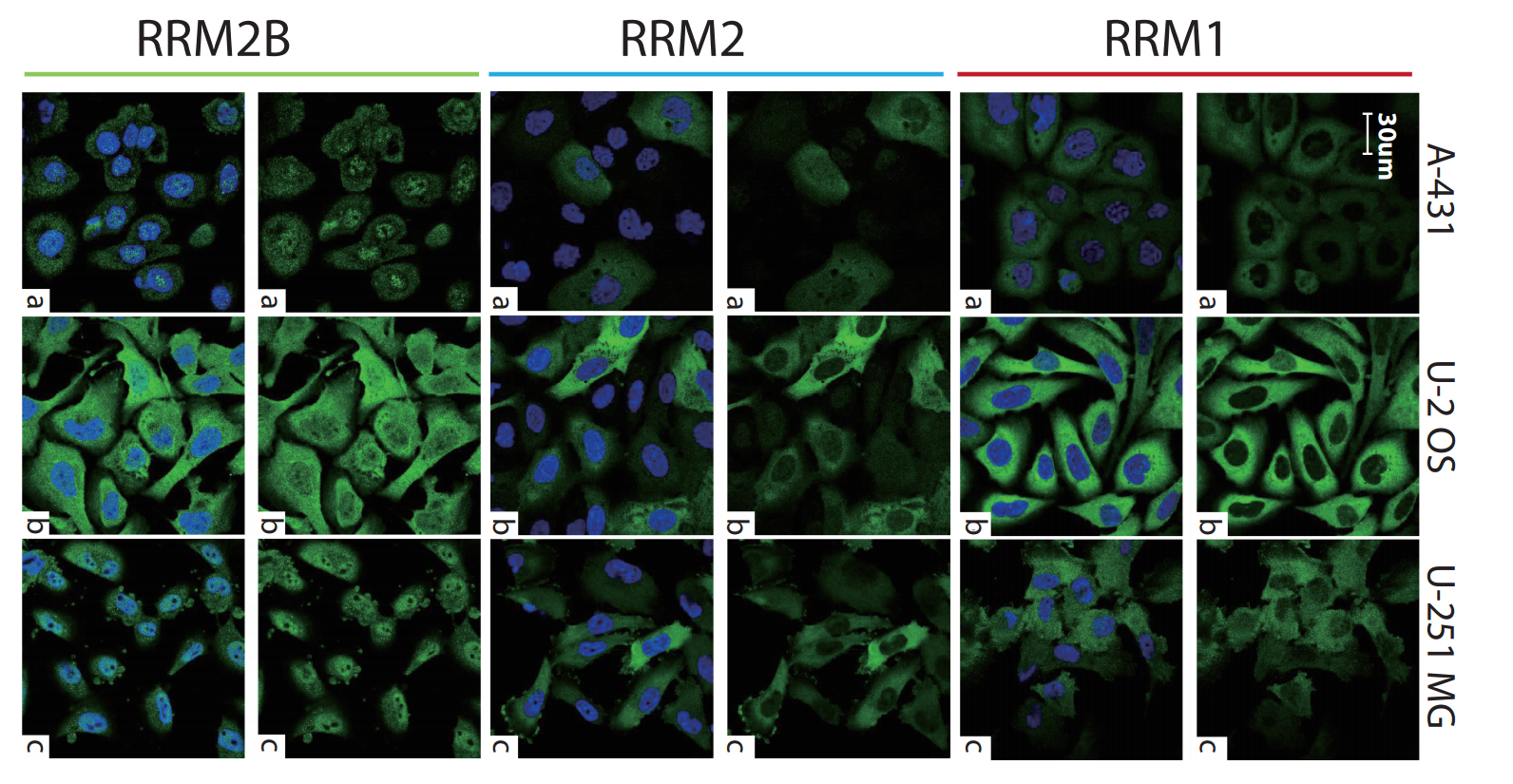


### Figure S3. The mRNA levels of *RRM1*, *RRM2* and *RRM2B* in LUAD and LUSC. Analysis were achieved from studies in Oncomine (for Lee et al., N = 138; for Bild et al., N = 111 ) and in TCGA (N = 1016). Green is for LUAD and blue is for LUSC. RSEM, RNA-Seq by Expectation Maximization. Student t test was performed for each comparison.


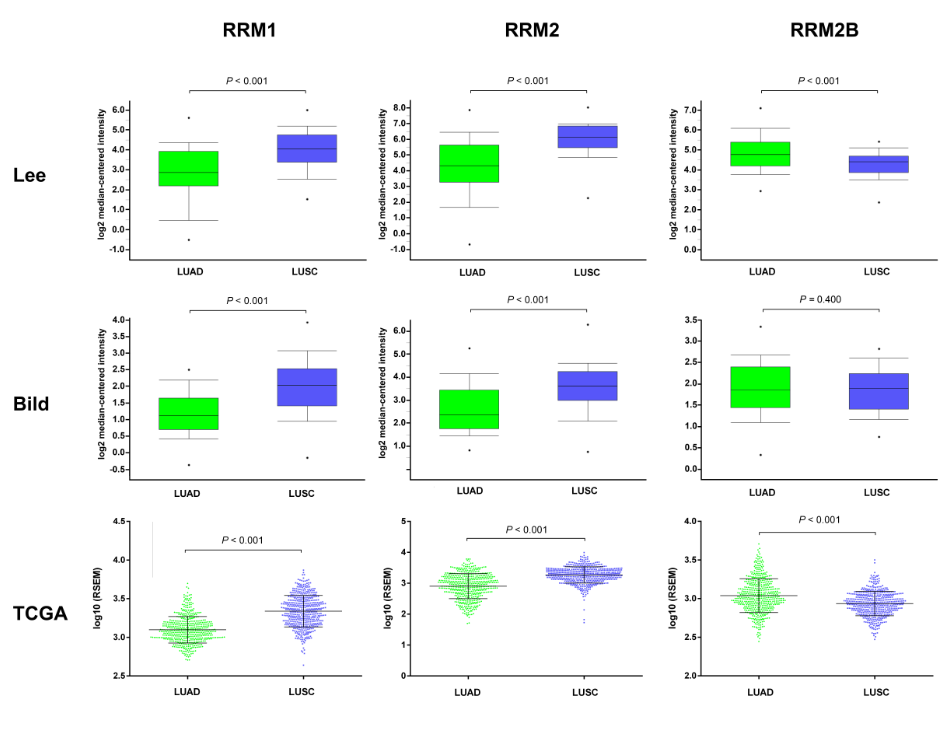


### Figure S4. Different prognostic values of *ERO1A* mRNA expression in LUAD, LUSC and LUSC + LUAD. According to the procedure provided by Uhlen et al.[62], we perform survival analysis (overall survival) of *ERO1A* expression in HPA. Best expression cutoffs for grouping the patients were selected by yielding the lowest log-rank *P*-value. The grouping results and log-rank *P*-values were shown in the panel of each Kaplan-Meier plot.


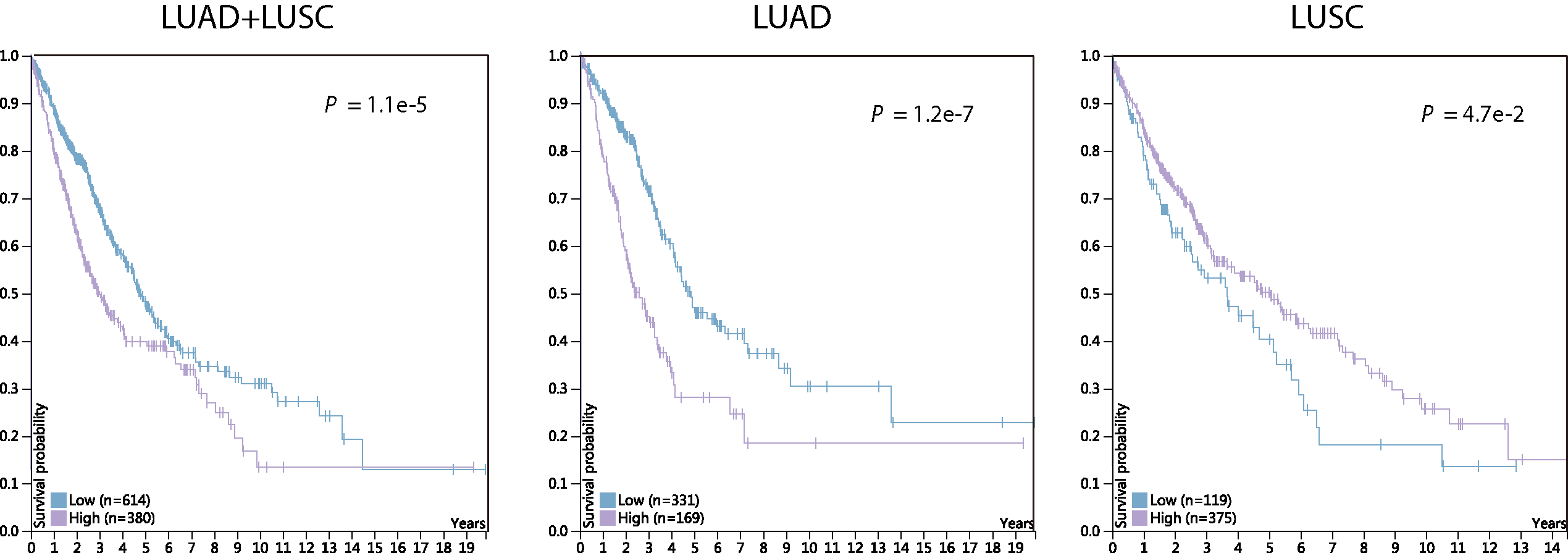


### Figure S5. Survival curves for the patients of LUAD and LUSC against the mRNA expression levels of RRM2B using optimal cutpoints. Based TCGA RNAseq data, we perform survival analysis (overall survival) of *RRM2B* expression in LUAD and LUSC through selecting optimal cutpoints provided by Uhlen et al.[62].


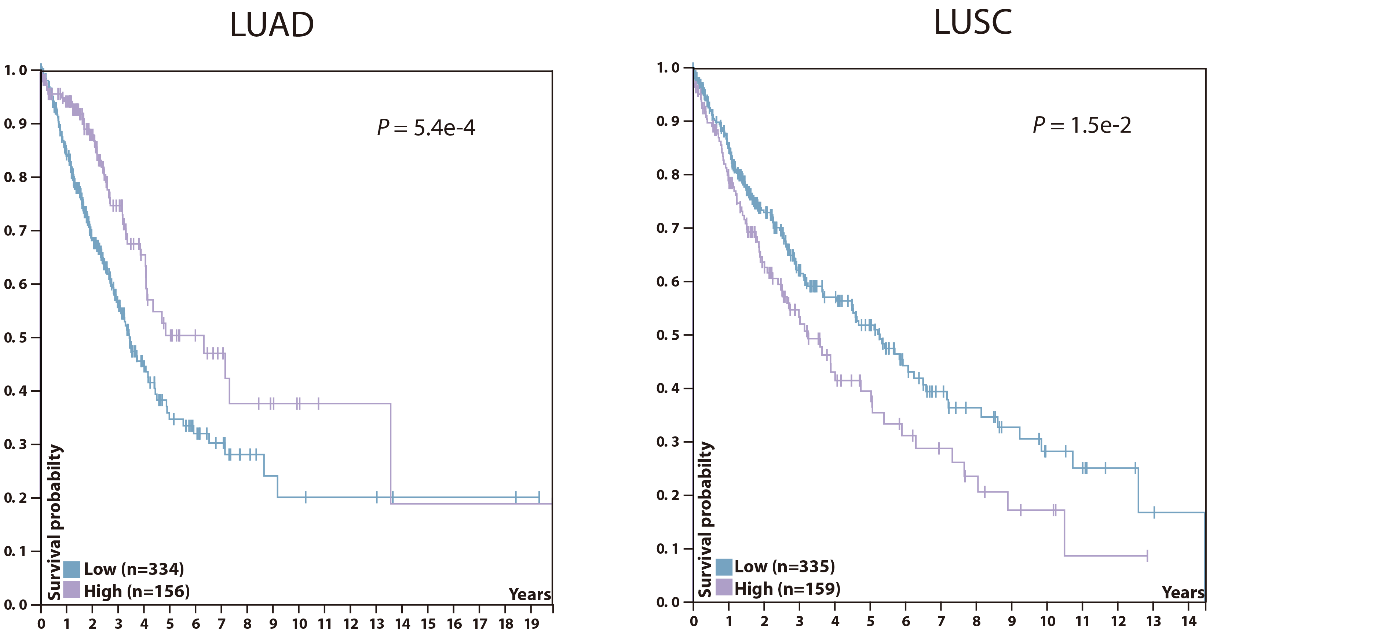

Supplement: Supplementary file 1 [file Data_Sheet_1.docx]
