# Supplementary material for: Integrative Analysis Reveals Across-Cancer Expression Patterns and Clinical Relevance of Ribonucleotide Reductase in Human Cancers
Source: Front Oncol. 2019 Oct 4;9:956. doi: 10.3389/fonc.2019.00956 (PMC6788385; doi:10.3389/fonc.2019.00956)
Supplement: Supplementary file 2 [file Data_Sheet_2.docx]

## Table S1. Demographics and clinicopathological characteristics of patients with LUSC and LUAD in ZJUC cohort

| Characteristics | LUSC (N = 97) | | LUAD (N = 114) | |
| --- | --- | --- | --- | --- |
|  | N (%) | | N (%) | |
| Age |  |  |  |  |
| ≤65 | 62 | (63.9) | 77 | (67.5) |
| >65 | 35 | (36.1) | 37 | (32.5) |
| Sex |  |  |  |  |
| Female | 1 | (1.0) | 58 | (50.9) |
| Male | 96 | (99.0) | 56 | (49.1) |
| Smoke |  |  |  |  |
| Yes | 92 | (94.8) | 38 | (33.3) |
| No | 5 | (5.2) | 76 | (66.7) |
| TNM Stage |  |  |  |  |
| I + II | 67 | (69.1) | 73 | (64.0) |
| III + IV | 30 | (30.9) | 41 | (36.0) |
| Infiltrating depth (T) |  |  |  |  |
| T1 + T2 | 71 | (73.2) | 101 | (88.6) |
| T3 + T4 | 26 | (26.8) | 13 | (11.4) |
| Lymph node metastasis (N) |  |  |  |  |
| N0 | 57 | (58.8) | 55 | (48.2) |
| N1 + N2 + N3 | 40 | (41.2) | 59 | (51.8) |
| Distant metastasis (M) |  |  |  |  |
| Absence | 91 | (93.8) | 107 | (93.9) |
| Presence | 6 | (6.2) | 7 | (6.1) |

## Table S2. Biological functions and cancer relations of *RRM2B* co-expressed genes

| LUAD |  |  |  |  |
| --- | --- | --- | --- | --- |
| Gene name | **Gene ID*** | **Gene function Summary** | **Cancer related studies** | **Significant functional enrichments(GO^#^ term)** |
| MYBL2 (MYB proto-oncogene like 2) | 4605 | The protein encoded by this gene, a member of the MYB family of transcription factor genes, is a nuclear protein involved in cell cycle progression. The encoded protein is phosphorylated by cyclin A/cyclin-dependent kinase 2 during the S-phase of the cell cycle and possesses both activator and repressor activities. It has been shown to activate the cell division cycle 2, cyclin D1, and insulin-like growth factor-binding protein 5 genes. | Liang HB, et al. found MYBL2 overexpression promotes GBC cell proliferation through the regulation of the cell cycle at the S and G2/M phase transitions [1].  Tao D, et al. found B-Myb promotes EMT and invasion of breast cancer cell [2]. | mitotic spindle assembly (GO: 0090307) |
| PIF1(PIF1 5'-to-3' DNA helicase) | 80119 | This gene encodes a DNA-dependent adenosine triphosphate (ATP)-metabolizing enzyme that functions as a 5' to 3' DNA helicase. The encoded protein can resolve G-quadruplex structures and RNA-DNA hybrids at the ends of chromosomes. It also prevents telomere elongation by inhibiting the actions of telomerase. | Gagou ME, et al. found Human PIF1 helicase supports DNA replication and cell growth under oncogenic-stress [3].  Gagou ME, et al. found suppression of apoptosis by PIF1 helicase in human tumor cells [4]. | DNA repair (GO term: 0006281) |
| KIFC1 (kinesin family member C1) | 3833 | A protein coding gene. GO annotations related to this gene include ATPase activity and microtubule motor activity. | Mittal et al. found KIFC1 predicts aggressive disease course in serous ovarian adenocarcinomas[5].  Pannu et al. found that KIFC1 overexpression fuels tumor progression via centrosome clustering-independent mechanisms in breast cancer patients[6]. | cell division (GO: 0051301);  mitotic spindle assembly (GO: 0090307);  mitotic sister chromatid segregation (GO: 0000070) |
| KIF18B (kinesin family member 18B) | 146909 | A protein coding gene. GO annotations related to this gene include ATPase activity and microtubule motor activity. | Few studies were reported | cell division (GO: 0051301);  mitotic sister chromatid segregation (GO: 0000070) |
| TROAP (trophinin associated protein) | 10024 | TROAP is a protein coding gene. Diseases associated with TROAP include Ectopic Pregnancy. It could be involved with bystin and trophinin in a cell adhesion molecule complex that mediates an initial attachment of the blastocyst to uterine epithelial cells at the time of the embryo implantation. | Few studies were reported |  |
| TICRR (TOPBP1 interacting checkpoint and replication regulator) | 90381 | A protein coding gene. Regulator of DNA replication and S/M and G2/M checkpoints | Few studies were reported | DNA repair (GO term: 0006281) |
| CDCA3 (cell division cycle associated 3) | 83461 | A protein coding gene. Its related pathways are cell cycle, cell division and protein ubiquitination. | Itzel et al. identified CDCA3 as novel driver genes in carcinogenesis[7].  Uchida et al. found that overexpression of CDCA3 promotes oral cancer progression by enhancing cell proliferation with prevention of G1 phase arrest[8]. | cell division (GO: 0051301) |
| PKMYT1 (protein kinase, membrane associated tyrosine/threonine 1) | 9088 | This gene encodes a member of the serine/threonine protein kinase family. The encoded protein is a membrane-associated kinase that negatively regulates the G2/M transition of the cell cycle by phosphorylating and inactivating cyclin-dependent kinase 1. | Liu L, et al. found PKMYT1 promoted the growth and motility of hepatocellular carcinoma cells [9]. |  |
| TACC3 (transforming acidic coiled-coil containing protein 3) | 10460 | This gene encodes a member of the transforming acidic coiled-coil protein family. The encoded protein is a motor spindle protein that may play a role in stabilization of the mitotic spindle. This protein may also play a role in growth a differentiation of certain cancer cells. | Li Q, et al. found overexpression of TACC3 is correlated with tumor aggressiveness and poor prognosis in prostate cancer [10].  Sun Y, et al. found overexpression of TACC3 reflects malignant characteristics and poor prognosis of Glioma [11]. | cell division (GO: 0051301) |
| C16ORF59 (TEDC2 tubulin epsilon and delta complex 2) | 80178 | TEDC2 (Tubulin Epsilon And Delta Complex 2) is a protein coding gene. | Few studies were reported |  |
| FCHO2 (FCH domain only 2) | 115548 | FCHO2 is a protein coding gene. Among its related pathways are Clathrin-mediated endocytosis and Vesicle-mediated transport. | Few studies were reported |  |
| TRIM23 (tripartite motif containing 23) | 373 | The protein encoded by this gene is a member of the tripartite motif (TRIM) family. The TRIM motif includes three zinc-binding domains, a RING, a B-box type 1 and a B-box type 2, and a coiled-coil region. This protein is also a member of the ADP ribosylation factor family of guanine nucleotide-binding family of proteins. Its carboxy terminus contains an ADP-ribosylation factor domain and a guanine nucleotide binding site, while the amino terminus contains a GTPase activating protein domain which acts on the guanine nucleotide binding site. The protein localizes to lysosomes and the Golgi apparatus. It plays a role in the formation of intracellular transport vesicles, their movement from one compartment to another, and phopholipase D activation. | Few studies were reported |  |
| EDA2R (ectodysplasin A2 receptor) | 60401 | The protein encoded by this gene is a type III transmembrane protein of the TNFR (tumor necrosis factor receptor) superfamily, and contains cysteine-rich repeats and a single transmembrane domain. This protein binds to the EDA-A2 isoform of ectodysplasin, which plays an important role in maintenance of hair and teeth. | Few studies were reported |  |
| CD302 (CD302 molecule) | 9936 | CD302 is a C-type lectin receptor involved in cell adhesion and migration, as well as endocytosis and phagocytosis | Kato M, et al. found Hodgkin's lymphoma cell lines express a fusion protein encoded by intergenically spliced mRNA for the multilectin receptor DEC-205 (CD205) and a novel C-type lectin receptor DCL-1 [12]. |  |
| PTCHD4 (patched domain containing 4) | 442213 | PTCHD4 is a protein coding gene. GO annotations related to this gene include hedgehog receptor activity. | Few studies were reported |  |
| LUSC |  |  |  |  |
| Gene Name | **Gene ID** | **Gene Function Summary** | **Cancer related studies** |  |
| LOC100130862 (also known as TRAM1, translocation associated membrane protein 1) |  | This gene encodes a multi-pass membrane protein that is part of the mammalian endoplasmic reticulum. The encoded protein influences glycosylation and facilitates the translocation of secretory proteins across the endoplasmic reticulum membrane by regulating which domains of the nascent polypeptide chain are visible to the cytosol during a translocational pause. |  |  |
| IMPA1 (inositol monophosphatase 1) | 3612 | This gene encodes an enzyme that dephosphorylates myo-inositol monophosphate to generate free myo-inositol, a precursor of phosphatidylinositol, and is therefore an important modulator of intracellular signal transduction via the production of the second messengers myoinositol 1,4,5-trisphosphate and diacylglycerol. | Few studies were reported |  |
| FAM91A1 (family with sequence similarity 91 member A1) | 157769 | FAM91A1 (Family With Sequence Similarity 91 Member A1) is a protein coding gene. | Few studies were reported |  |
| EFR3A (EFR3 homolog A) | 23167 | The protein encoded by this gene is part of a complex that plays a role in maintaining an active pool of phosphatidylinositol 4-kinase (PI4K) at the plasma membrane. This protein is thought to be a peripheral membrane protein that associates with the plasma membrane through palmitoylation. Studies indicate that this gene product plays a role in controlling G protein-coupled receptor (GPCR) activity by affecting receptor phosphorylation. | Few studies were reported |  |
| DPY19L4 (dpy-19 like 4) | 286148 | DPY19L4 is a protein coding gene. GO annotations related to this gene include transferase activity, transferring glycosyl groups and mannosyltransferase activity. | Few studies were reported |  |
| AZIN1 (antizyme inhibitor 1) | 51582 | The protein encoded by this gene belongs to the antizyme inhibitor family, which plays a role in cell growth and proliferation by maintaining polyamine homeostasis within the cell. Antizyme inhibitors are homologs of ornithine decarboxylase (ODC, the key enzyme in polyamine biosynthesis) that have lost the ability to decarboxylase ornithine; however, retain the ability to bind to antizymes. Antizymes negatively regulate intracellular polyamine levels by binding to ODC and targeting it for degradation, as well as by inhibiting polyamine uptake. Antizyme inhibitors function as positive regulators of polyamine levels by sequestering antizymes and neutralizing their effect. This gene encodes antizyme inhibitor 1, the first member of this gene family that is ubiquitously expressed, and is localized in the nucleus and cytoplasm. Overexpression of antizyme inhibitor 1 gene has been associated with increased proliferation, cellular transformation and tumorigenesis. Gene knockout studies showed that homozygous mutant mice lacking functional antizyme inhibitor 1 gene died at birth with abnormal liver morphology. RNA editing of this gene, predominantly in the liver tissue, has been linked to the progression of hepatocellular carcinoma. | Hu X, *et al*. found RNA editing of AZIN1 induces the malignant progression of non-small-cell lung cancers [13]. |  |
| ATP6V1C1 (ATPase H+ transporting V1 subunit C1) | 528 | This gene encodes a component of vacuolar ATPase (V-ATPase), a multisubunit enzyme that mediates acidification of intracellular compartments of eukaryotic cells. V-ATPase dependent acidification is necessary for such intracellular processes as protein sorting, zymogen activation, receptor-mediated endocytosis, and synaptic vesicle proton gradient generation. | Cai M, et al. found ATP6V1C1 may regulate filament actin arrangement in breast cancer cells [14]. |  |
| UBE2W (ubiquitin conjugating enzyme E2 W) | 55284 | This gene encodes a nuclear-localized ubiquitin-conjugating enzyme (E2) that, along with ubiquitin-activating (E1) and ligating (E3) enzymes, coordinates the addition of a ubiquitin moiety to existing proteins. The encoded protein promotes the ubiquitination of Fanconi anemia complementation group proteins and may be important in the repair of DNA damage. | Few studies were reported |  |
| SLC25A32 (solute carrier family 25 member 32) | 81034 | This gene encodes a member of the P(I/L)W subfamily of mitochondrial carrier family transport proteins. The encoded protein transports folate across the inner mitochondrial membrane. | Few studies were reported |  |
| RNF139 (ring finger protein 139) | 11236 | The protein encoded by this gene is a multi-membrane spanning protein containing a RING-H2 finger. This protein is located in the endoplasmic reticulum, and has been shown to possess ubiquitin ligase activity. This gene was found to be interrupted by a t(3:8) translocation in a family with hereditary renal and non-medulary thyroid cancer. | Wang L, et al. found RNF139 inhibits the progression of tongue cancer[15]. | protein destabilization (GO: 0031648);  ERAD pathway (GO: 0036503) |
| PRO2577 (also known as DERL1, derlin 1) | 79139 | The protein encoded by this gene is a member of the derlin family. Members of this family participate in the ER-associated degradation response and retrotranslocate misfolded or unfolded proteins from the ER lumen to the cytosol for proteasomal degradation. This protein recognizes substrate in the ER and works in a complex to retrotranslocate it across the ER membrane into the cytosol. This protein may select cystic fibrosis transmembrane conductance regulator protein (CFTR) for degradation as well as unfolded proteins in Alzheimer's disease. Alternative splicing results in multiple transcript variants that encode different protein isoforms.  https://www.ncbi.nlm.nih.gov/gene/79139 |  | protein destabilization (GO: 0031648);  ERAD pathway (GO: 0036503) |
| PLEKHF2 (pleckstrin homology and FYVE domain containing 2) | 79666 | PLEKHF2 (Pleckstrin Homology And FYVE Domain Containing 2) is a protein coding gene, may play a role in early endosome fusion upstream of RAB5, hence regulating receptor trafficking and fluid-phase transport. Enhances cellular sensitivity to TNF-induced apoptosis | Few studies were reported |  |
| MTDH (metadherin) | 92140 | MTDH (Metadherin) is a protein coding gene. Diseases associated with MTDH include Nervous System Disease and Central Nervous System Disease. Among its related pathways are Cytoskeleton remodeling Regulation of actin cytoskeleton by Rho GTPases and Adhesion. | Gu C, et al. found MTDH is an oncogene in multiple myeloma, which is suppressed by Bortezomib treatment [16].  Li WF, et al. Lentiviral-mediated short hairpin RNA knockdown of MTDH inhibits cell growth and induces apoptosis by regulating the PTEN/AKT Pathway in hepatocellular carcinoma [17]. |  |

| Common in LUAD and LUSC |  |  |  |
| --- | --- | --- | --- |
| Gene Name | **Gene ID** | **Gene Function Summary** | **Cancer related studies** |
| PRO1071 (CPNE3 copine 3) | 8895 | Calcium-dependent membrane-binding proteins may regulate molecular events at the interface of the cell membrane and cytoplasm. This gene encodes a protein which contains two type II C2 domains in the amino-terminus and an A domain-like sequence in the carboxy-terminus. The A domain mediates interactions between integrins and extracellular ligands. | Fu L, et al. found high expression of CPNE3 predicts adverse prognosis in acute myeloid leukemia [18].  Lin HC, et al. found quantitative proteomic analysis identifies CPNE3 as a novel metastasis-promoting gene in NSCLC [19]. |

*derived from: <https://www.ncbi.nlm.nih.gov/gene>; ^#^Gene Ontology.

Note: all information was achieved from GENE (https://www.ncbi.nlm.nih.gov/gene) and GeneCards (<http://www.genecards.org>)

# References:

1. Liang HB, Cao Y, Ma Q, Shu YJ, Wang Z, Zhang F, Ye YY, Li HF, Xiang SS, Song XL, et al: **MYBL2 is a Potential Prognostic Marker that Promotes Cell Proliferation in Gallbladder Cancer.** *Cell Physiol Biochem* 2017, **41:**2117-2131.

2. Tao D, Pan Y, Jiang G, Lu H, Zheng S, Lin H, Cao F: **B-Myb regulates snail expression to promote epithelial-to-mesenchymal transition and invasion of breast cancer cell.** *Med Oncol* 2015, **32:**412.

3. Gagou ME, Ganesh A, Phear G, Robinson D, Petermann E, Cox A, Meuth M: **Human PIF1 helicase supports DNA replication and cell growth under oncogenic-stress.** *Oncotarget* 2014, **5:**11381-11398.

4. Gagou ME, Ganesh A, Thompson R, Phear G, Sanders C, Meuth M: **Suppression of apoptosis by PIF1 helicase in human tumor cells.** *Cancer Res* 2011, **71:**4998-5008.

5. Itzel T, Scholz P, Maass T, Krupp M, Marquardt JU, Strand S, Becker D, Staib F, Binder H, Roessler S, et al: **Translating bioinformatics in oncology: guilt-by-profiling analysis and identification of KIF18B and CDCA3 as novel driver genes in carcinogenesis.** *Bioinformatics* 2015, **31:**216-224.6. Uchida F, Uzawa K, Kasamatsu A, Takatori H, Sakamoto Y, Ogawara K, Shiiba M, Tanzawa H, Bukawa H: **Overexpression of cell cycle regulator CDCA3 promotes oral cancer progression by enhancing cell proliferation with prevention of G1 phase arrest.** *BMC Cancer* 2012, **12:**321.

7. Mittal K, Choi DH, Klimov S, Pawar S, Kaur R, Mitra AK, Gupta MV, Sams R, Cantuaria G, Rida PCG, Aneja R: **A centrosome clustering protein, KIFC1, predicts aggressive disease course in serous ovarian adenocarcinomas.** *J Ovarian Res* 2016, **9:**17.

8. Pannu V, Rida PC, Ogden A, Turaga RC, Donthamsetty S, Bowen NJ, Rudd K, Gupta MV, Reid MD, Cantuaria G, et al: **HSET overexpression fuels tumor progression via centrosome clustering-independent mechanisms in breast cancer patients.** *Oncotarget* 2015, **6:**6076-6091.

9. Liu L, Wu J, Wang S, Luo X, Du Y, Huang D, Gu D, Zhang F: **PKMYT1 promoted the growth and motility of hepatocellular carcinoma cells by activating beta-catenin/TCF signaling.** *Exp Cell Res* 2017, **358:**209-216.

10. Li Q, Ye L, Guo W, Wang M, Huang S, Peng X: **Overexpression of TACC3 is correlated with tumor aggressiveness and poor prognosis in prostate cancer.** *Biochem Biophys Res Commun* 2017, **486:**872-878.

11. Sun Y, Tian Y, Wang GZ, Zhao SH, Han B, Li YL, Jiang CL: **Overexpression of Transforming Acidic Coiled CoilContaining Protein 3 Reflects Malignant Characteristics and Poor Prognosis of Glioma.** *Int J Mol Sci* 2017, **18**.

12. Kato M, Khan S, Gonzalez N, O'Neill BP, McDonald KJ, Cooper BJ, Angel NZ, Hart DN: **Hodgkin's lymphoma cell lines express a fusion protein encoded by intergenically spliced mRNA for the multilectin receptor DEC-205 (CD205) and a novel C-type lectin receptor DCL-1.** *J Biol Chem* 2003, **278:**34035-34041.

13. Hu X, Chen J, Shi X, Feng F, Lau KW, Chen Y, Chen Y, Jiang L, Cui F, Zhang Y, et al: **RNA editing of AZIN1 induces the malignant progression of non-small-cell lung cancers.** *Tumour Biol* 2017, **39:**1010428317700001.

14. Cai M, Liu P, Wei L, Wang J, Qi J, Feng S, Deng L: **Atp6v1c1 may regulate filament actin arrangement in breast cancer cells.** *PLoS One* 2014, **9:**e84833.

15. Wang L, Yin W, Shi C: **E3 ubiquitin ligase, RNF139, inhibits the progression of tongue cancer.** *BMC Cancer* 2017, **17:**452.

16. Gu C, Feng L, Peng H, Yang H, Feng Z, Yang Y: **MTDH is an oncogene in multiple myeloma, which is suppressed by Bortezomib treatment.** *Oncotarget* 2016, **7:**4559-4569.

17. Li WF, Ou Q, Dai H, Liu CA: **Lentiviral-Mediated Short Hairpin RNA Knockdown of MTDH Inhibits Cell Growth and Induces Apoptosis by Regulating the PTEN/AKT Pathway in Hepatocellular Carcinoma.** *Int J Mol Sci* 2015, **16:**19419-19432.

18. Fu L, Fu H, Qiao J, Pang Y, Xu K, Zhou L, Wu Q, Li Z, Ke X, Xu K, Shi J: **High expression of CPNE3 predicts adverse prognosis in acute myeloid leukemia.** *Cancer Sci* 2017, **108:**1850-1857.

19. Lin HC, Zhang FL, Geng Q, Yu T, Cui YQ, Liu XH, Li J, Yan MX, Liu L, He XH, et al: **Quantitative proteomic analysis identifies CPNE3 as a novel metastasis-promoting gene in NSCLC.** *J Proteome Res* 2013, **12:**3423-3433.

## Table S3. Common cancer-omics databases and related analytic tools

| Name | Data type | Data source | Cancer type / sample size | Function / Characteristics | Web server | Refs |
| --- | --- | --- | --- | --- | --- | --- |
| COSMIC  (Catalogue of Somatic Mutations in Cancer) | mutation | WGS | > 1.5 million individual mutations in > 25,000 genes in almost 950,000 samples from almost all cancers | Largest and most comprehensive resource for exploring the impact of somatic mutations in human cancer. It includes several distinct projects: COSMIC (an expert-curated database of somatic mutations), Cell Lines Project (mutation profiles of over 1,000 cell lines used in cancer research), COSMIC-3D (An interactive view of cancer mutations in the context of 3D structures), Cancer Gene Census (A catalogue of genes with mutations that are causally implicated in cancer) | http://cancer.sanger.ac.uk/cosmic | [2] |
| Oncomine | mRNA | Gene microarray | > 20 common tumor types, > 700 datasets and > 86,000 tumor samples | Differential expression analysis, co-expression analysis, molecular concepts analysis, and interaction network analysis. | https://www.oncomine.org | [6] |
| KM-plotter  (Kaplan-Meier plotter) | mRNA  miRNA | Gene microarray  RNA-seq | Breast, ovarian, lung, liver and gastric cancer, which includes > 10000 samples | Online survival analysis software to assess the prognostic value of specific gene using transcriptomic data. | http://kmplot.com/analysis | [8] |
| GEPIA  (Gene Expression Profiling Interactive Analysis) | mRNA | RNA-seq | 33 tumor types, > 9700 tumor and > 8500 normal samples | Provides functions such as tumor/normal differential expression analysis, profiling according to cancer types or pathological stages, patient survival analysis, similar gene detection, correlation analysis and dimensionality reduction analysis. | http://gepia.cancer-pku.cn/ | [29] |
| TCPA  (The Cancer Proteome Atlas) | protein | RPPA | Contains expression levels of key cancer proteins; > 8,000 samples of 32 cancer types from TCGA and other independent patient cohorts; > 650 independent cell lines across 19 lineages. | It enables users to perform various common protein-centered analyses, including correlation, differential expression, patient survival, and drug sensitivity analyses. It provides a unique opportunity to validate the findings from TCGA data and identify model cell lines for functional investigation. | http://tcpaportal.org/tcpa | [5] |
| HPM  (Human Proteome Map) | protein | Protein sequencing | Focus on normal tissues: proteins encoded by > 17,000 genes were identified in 17 adult tissues, 6 primary hematopoietic cells and 7 fetal tissues from 85 samples. | A unique and comprehensive strategy for proteogenomic analysis enabled us to discover a number of novel protein-coding regions, which includes translated pseudogenes, non-coding RNAs and upstream open reading frames. It will complement available human genome and transcriptome data to accelerate biomedical research in health and disease. | http://www.humanproteomemap.org | [30] |
| TCGA  (The Cancer Genome Atlas) | Mutation  CNV  Methylation  mRNA  miRNA | WES  Genotyping Array  Methylation Array  RNA-seq  miRNA-seq | 11000 cancer patients from 33 common types of Cancer | Provides multi-omics data to generate comprehensive, multi-dimensional maps of the key genomic changes. | https://cancergenome.nih.gov,  The NCI's Genomic Data Commons (GDC, https://portal.gdc.cancer.gov),  cBio Cancer Genomics Portal (cBioPortal, www.cbioportal.org),  Gene Exchange Profiling Interactive (Gepia, http://gepia.cancer-pku.cn),  Firehose (http://gdac.broadinstitute.org) | [3] |
| HPA  (The Human Protein Atlas) | mRNA  protein | RNA-seq  IHC  IFC | 20 different common tumor types and 17000 protein-code genes | Composed of three atlas:  (1) The Human Pathology Atlas analyzes the genome-wide transcriptome of the protein-coding genes of 17 major cancer types with respect to clinical outcome.  (2) The Human Cell Atlas resolves the spatial distribution of the human proteome at a subcellular level.  (3) The Human Tissue Atlas allows exploring the tissue-restricted expression of the human proteome and transcriptome in all major tissues and organs. | https://www.proteinatlas.org | [9] |
| cBioPortal  (cBio Cancer Genomics Portal) | Mutation  CNA  mRNA  miRNA  protein | WGS  WXS  RNAseq  RPPA | > 30 tumor types and 225 independent tumor studies | Provides visualization and analysis of somatic mutations, copy number alterations, mRNA expression and survival plots of interest genes. | http://www.cbioportal.org/ | [7] |
| ICGC  (International Cancer Genome Consortium) | mutation (primary)  mRNA  miRNA | WGS  WXS  RNA-seq  miRNA-seq | 22 cancer types, > 24000 donors, > 774600 somatic mutations | Reveals the repertoire of oncogenic mutations, uncover traces of the mutagenic influences, define clinically relevant subtypes for prognosis and therapeutic management, and enable the development of new cancer therapies. | https://icgc.org | [4] |
| GTEx  (Genotype-Tissue Expression) | Genetic variation  mRNA | WGS  RNA-seq | multiple human tissues (i.e. brain, heart, lung, breast, skin and whole blood etc.) from ~960 donors and over 30,000 samples | Study the relationship between genetic variation and gene expression in multiple human tissues. GTEx is also examining sex-based differences in how genes are turned on and off and how they are regulated. GTEx resources are valuable tools for exploring the genetic basis of complex human diseases, such as cancer. | https://www.gtexportal.org | [31] |
| CCLE  (Cancer Cell Line Encyclopedia) | mutation  CNV  methylation  mRNA  protein | Massively parallel sequencing/  SNP arrays  RRBS  Gene microarray/  RNA-seq  RPPA | Large-scale genomic data set for over 1100 human cancer cell lines, together with pharmacological profiling of 24 compounds across ∼500 of these lines. | Explore the expression of interested genes in specific cancer cell lines. In addition, this collection allowed identification of genetic, lineage, and gene-expression-based predictors of drug sensitivity. | https://portals.broadinstitute.org/ccle | [32] |

WGS, Whole-genome sequencing; WES, Whole exome sequencing; RPPA, Reverse-phase protein arrays; RRBS, Reduced representation bisulfite sequencing; IHC, Immunohistochemistry; IFC, Immunofluorescence

## Table S4. The mutation of RRM1, RRM2 and RRM2B in public available databases.

| **Database** | **Mutation type** | ***RRM1*** | ***RRM2*** | ***RRM2B*** |
| --- | --- | --- | --- | --- |
| COSMIC  (v83) | Substitution nonsense | 3 | 2 | 3 |
|  | Substitution missense | 97 | 52 | 67 |
|  | Substitution synonymous | 33 | 24 | 13 |
|  | Other | 6 | 4 | 5 |
|  | Mutated samples（%） | 139(0.43) | 82(0.26) | 88(0.27) |
|  | Mutation count>3 | R499C/H | NO | P308L/S. F323L |
|  | Samples Tested | 32255 | 32062 | 32098 |
| cBioPortal | Missense | 109 | 86 | 70 |
|  | Truncating | 14 | 14 | 8 |
|  | Inframe | 0 | 0 | 0 |
|  | Other | 0 | 0 | 0 |
|  | Mutated samples（%） | 123(0.30) | 100(0.24) | 78(0.19) |
|  | sequenced cases | 41603 | 41603 | 41603 |

Note: data was derived from COSMIC (v83, released 07-NOV-17) and cBioPortal (Version 1.11.3).

## Table S5. The differential expressions of RR genes in Oncomine

|  | | Aye *et al.* (Done on Nov, 2013)* | | | This study (Done on Mar, 2019)# | | |
| --- | --- | --- | --- | --- | --- | --- | --- |
|  |  | Differentially expressed/total study | | % | Differentially expressed/total study | | % |
| *RRM1* | Overexpression | 30/170 | 17.6 | | 86/358 | 24.0 | |
|  | Underexpression | 6/170 | 3.5 | | 30/358 | 8.4 | |
| *RRM2* | Overexpression | 73168 | 43.5 | | 136/355 | 38.3 | |
|  | Underexpression | 7/168 | 4.2 | | 16/355 | 4.5 | |
| *RRM2B* | Overexpression | 5/96 | 5.2 | | 22/209 | 10.5 | |
|  | Underexpression | 0/96 | 0 | | 10/209 | 4.8 | |

* The thresholds for differentially expressed genes are set to “concept filter = cancers vs. normal, data type = mRNA, and gene rank ≤ 10 %” in Aye *et al.*,

# The thresholds for differentially expressed genes are set to “concept filter = cancers vs. normal, data type = mRNA, P-value < 0.05, and gene rank ≤ 10 %”in this study.
